# Influence of mechanical and TGF-β3 stimulation on the tenogenic differentiation of tonsil-derived mesenchymal stem cells

**DOI:** 10.1186/s12860-021-00400-7

**Published:** 2022-01-15

**Authors:** Jaeyeon Wee, Hyang Kim, Sang-Jin Shin, Taeyong Lee, Seung Yeol Lee

**Affiliations:** 1 School of Medicine, CHA University, Pocheon, South Korea; 2 New Horizon Biomedical Engineering Institute, Myongji Hospital, Goyang, South Korea; 3 Department of Orthopaedic Surgery, Ewha Womans University Seoul Hospital, Seoul, South Korea; 4 Division of Mechanical and Biomedical Engineering, Ewha Womans University, Seoul, South Korea; 5 Department of Orthopaedic Surgery, Myongji Hospital, Hanyang University College of Medicine, 55, Hwasu-ro 14beon-gil, Deogyang-gu, Goyang, Gyeonggi 10475 South Korea

**Keywords:** Tendinopathy, Tonsil-derived mesenchymal cells, Mechanical strain, TGF-β3

## Abstract

**Background:**

Organogenesis from tonsil-derived mesenchymal cells (TMSCs) has been reported, wherein tenogenic markers are expressed depending on the chemical stimulation during tenogenesis. However, there are insufficient studies on the mechanical strain stimulation for tenogenic cell differentiation of TMSCs, although these cells possess advantages as a cell source for generating tendinous tissue. The purpose of this study was to investigate the effects of mechanical strain and transforming growth factor-beta 3 (TGF-β3) on the tenogenic differentiation of TMSCs and evaluate the expression of tendon-related genes and extracellular matrix (ECM) components, such as collagen.

**Results:**

mRNA expression of tenogenic genes was significantly higher when the mechanical strain was applied than under static conditions. Moreover, mRNA expression of tenogenic genes was significantly higher with TGF-β3 treatment than without. mRNA expression of osteogenic and chondrogenic genes was not significantly different among different mechanical strain intensities. In cells without TGF-β3 treatment, double-stranded DNA concentration decreased, while the amount of normalized collagen increased as the intensity of mechanical strain increased.

**Conclusions:**

Mechanical strain and TGF-β3 have significant effects on TMSC differentiation into tenocytes. Mechanical strain stimulates the differentiation of TMSCs, particularly into tenocytes, and cell differentiation, rather than proliferation. However, a combination of these two did not have a synergistic effect on differentiation. In other words, mechanical loading did not stimulate the differentiation of TMSCs with TGF-β3 supplementation. The effect of mechanical loading with TGF-β3 treatment on TMSC differentiation can be manipulated according to the differentiation stage of TMSCs. Moreover, TMSCs have the potential to be used for cell banking, and compared to other mesenchymal stem cells, they can be procured from patients via less invasive procedures.

## Background

Tendinopathy is a chronic condition that hinders normal movement and causes inconvenience in daily life [1]. Upon injury of the tendon, a full return to its original state in terms of its structure and strength is often not achieved despite tendon repair surgery [2]. Acute and chronic tendon injuries can result in a decline in the quality of life. As society ages, the incidence of tendinopathy increases [3], and thus appropriate treatment for tendon injury should be developed.

Currently, direct repair or tendon transfer is predominantly used to treat tendinopathy [4, 5]. However, these remedies have some disadvantages as follows: the repair site consists of fibrous tissue rather than the original tissue post-surgery, which may cause fibrosis; the healing potency is low to eliminate scarring; and adhesion with other connective tissues is low [6]. To overcome these drawbacks, mesenchymal stem cells (MSCs) have been developed for use in tissue engineering. MSCs can be derived from many sites, such as adipose tissue, bone marrow, and cord blood [7]. Furthermore, MSCs are multipotent and can differentiate into tendons under appropriate culture conditions. Moreover, MSCs have shown promising potential for tendon repair in vitro and in animal studies [8, 9].

According to a previous study, adipose tissue-derived MSCs overexpressing scleraxis (SCX) have increased tenogenic gene expression and affect tendon cell differentiation [10]. Furthermore, the uniaxial strain on MSCs from the bone marrow is positively correlated with tendon matrix generation [11]. MSCs can be tenogenic under mechanical strain conditions and chemical stimulation [12, 13]. However, these stem cell-harvesting procedures are invasive and harmful, and donor site morbidity and the derived yield from single cells are limited [14]. Tonsil-derived mesenchymal cells (TMSCs) are considered waste tissues from tonsillectomy, although tissue banking is possible [15]. Organogenesis from TMSCs has been reported, wherein tenogenic markers are expressed depending on the chemical stimulation during tenogenesis [16]. However, there are insufficient studies on mechanical strain stimulation for tenogenic cell differentiation of TMSCs, although these cells possess advantages as a source for generating tendinous tissue.

The purpose of this study was to investigate the effects of mechanical strain and transforming growth factor-beta 3 (TGF-β3) on the tenogenic differentiation of TMSCs and evaluate the expression of tendon-related genes and extracellular matrix (ECM) components, such as collagen. It was hypothesized that mechanical strain and TGF-β3 induced tendon-related gene expression and increased ECM components.

## Results

**Fig. 1:**
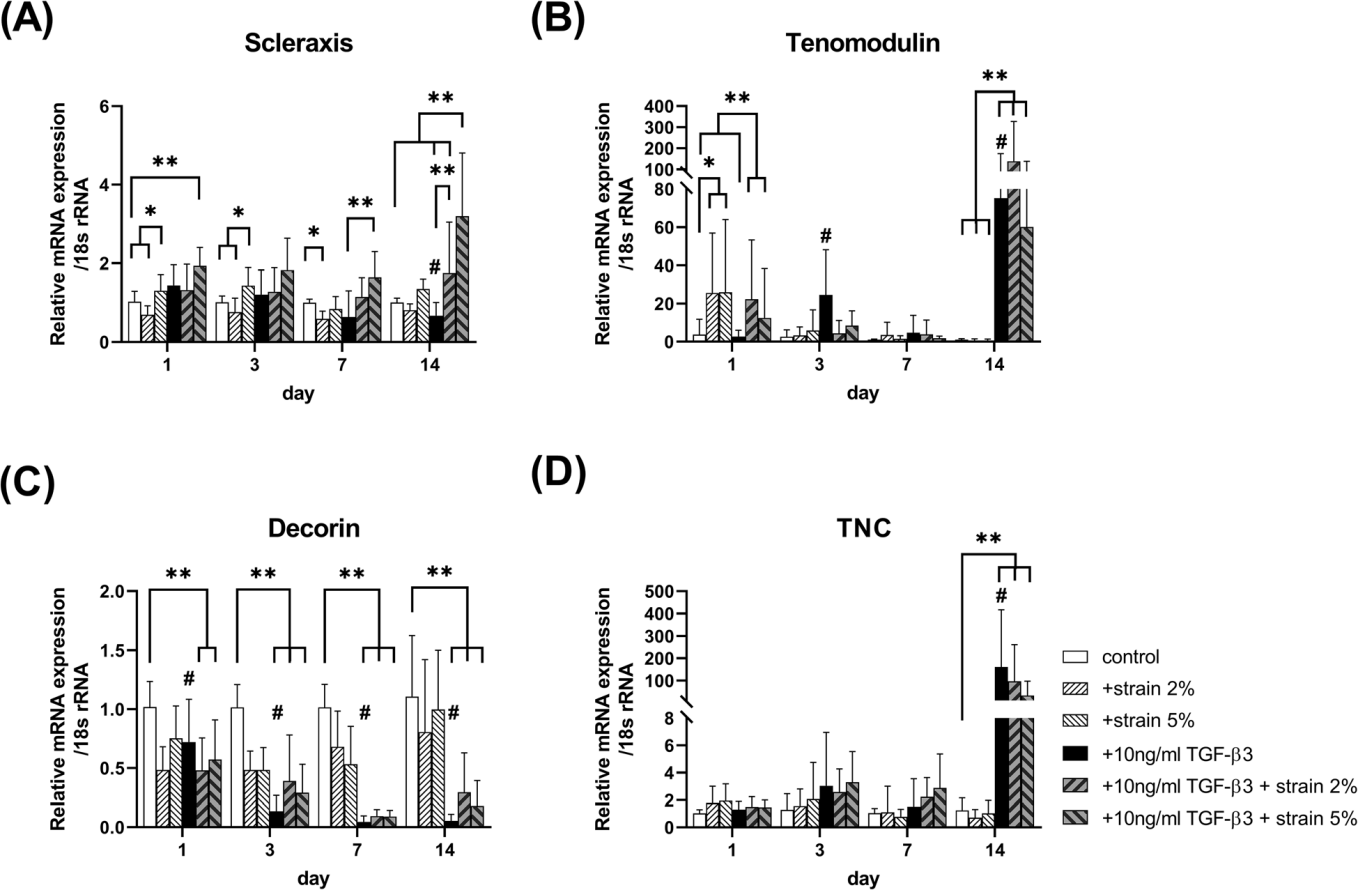
mRNA expression of tenogenic genes (**A**) *scleraxis*, (**B**) *tenomodulin*, (**C**) *decorin*, and (**D**) *TNC* was measured using qRT-PCR after 1, 3, 7, and 14 days under mechanical strain without or with 10 ng/ml TGF-β3. Data shown are mean fold static condition group ± SEM at each time point. Significant differences are represented as follows (*p < 0.05)*: static control vs strain groups without TGF-β3(*), between all groups (**), and without vs with 10 ng/ml TGF-β3 under static control (#)

**Fig. 2:**
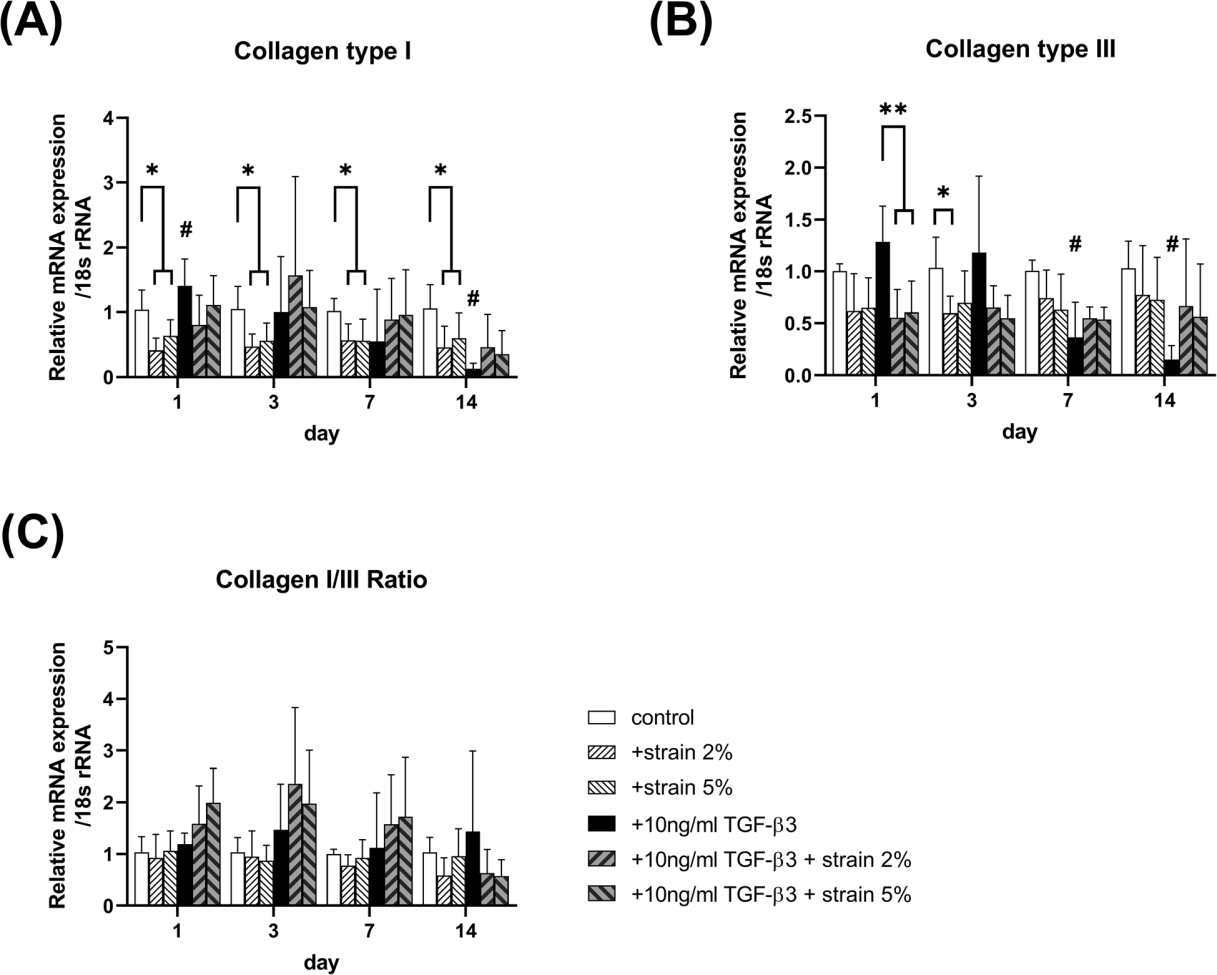
mRNA expression of collagen genes (**A**) *COLI*, (**B**) *COLIII*, and (**C**) *COLI/III* measured using qRT-PCR after 1, 3, 7, and 14 days under mechanical strain without or with 10 ng/ml TGF-β3. Data shown are mean fold static condition group ± SEM at each time point

When TMSCs were treated with TGF-β3 without mechanical stimulation, the mRNA expression of tenomodulin (*TNMD*) was increased on day 3 compared to that in the untreated group (*p = 0.012*; Fig.1B). Especially, in the TGF-β3 group, the mRNA expression levels of *TNMD* (*p = 0.033*) and tenascin-C (*TNC*) (*p = 0.054*; Fig.1D) on day 14 were significantly higher than those of the TGF-β3 untreated group. However, TGF-β3 decreased the mRNA expression of *SCX* (*p = 0.001*; Fig.1A) and collagen type 1 (*COL1*) (*p < 0.001*; Fig.2A) on day 14 as well as that of *COL3* on days 7 and 14 (*p < 0.001*; Fig.2B). Moreover, the mRNA expression of decorin (*DCN*) in TMSCs was decreased significantly at all time points by TGF treatment (*p = 0.023* on day 3 and *p < 0.001* on other days; Fig.1C).

Mechanical strain stimulated the mRNA expression of tenogenic marker genes in TGF-β3-untreated TMSCs. The mRNA expression of *SCX* under 2% strain without TGF-β3 treatment was significantly lesser than that under static control before day 7 (*p < 0.001*), and the mRNA expression under 5% strain in the TGF-β3-untreated strain group was the highest on days 1 and 3 (*p = 0.008 and p < 0.001*, respectively; Fig.1A). In addition, the mRNA expression of *TNMD* was increased in the strain group without TGF-β3 compared to that observed under static control on day 1, regardless of the strain intensity (*p < 0.05*; Fig.1B). There was no statistically significant difference in the mRNA expression of *DCN* and *TNC* (Fig.1C and 1D). The mRNA expression of *COL1* was decreased in the strain group without TGF-β3 compared to that observed under static control over the whole experimental period (*p < 0.001*; Fig.2A). *COL3* expression was decreased in the 2% strain group on day 3(*p = 0.043*; Fig.2B), and there was no statistically significant difference in the COL1/3 ratio between the strain groups without TGF-β3 (Fig.2C).

In the TGF-β3-treated strain TMSC group, the mRNA expression of *SCX*, *TNMD*, and *TNC* was significantly increased compared to that in the group without TGF-β3 on day 14 (*p < 0.05*), regardless of the strain intensity, but there was no synergistic effect between TGF-β3 and mechanical stimulation. Further, the mRNA expression of *DCN* was not further reduced by simultaneous treatment with TGF-β3 and mechanical stimulation (Fig. 1). The same results were obtained for the mRNA expression of *COL1* and *COL3* (Fig.2).

**Fig. 3:**
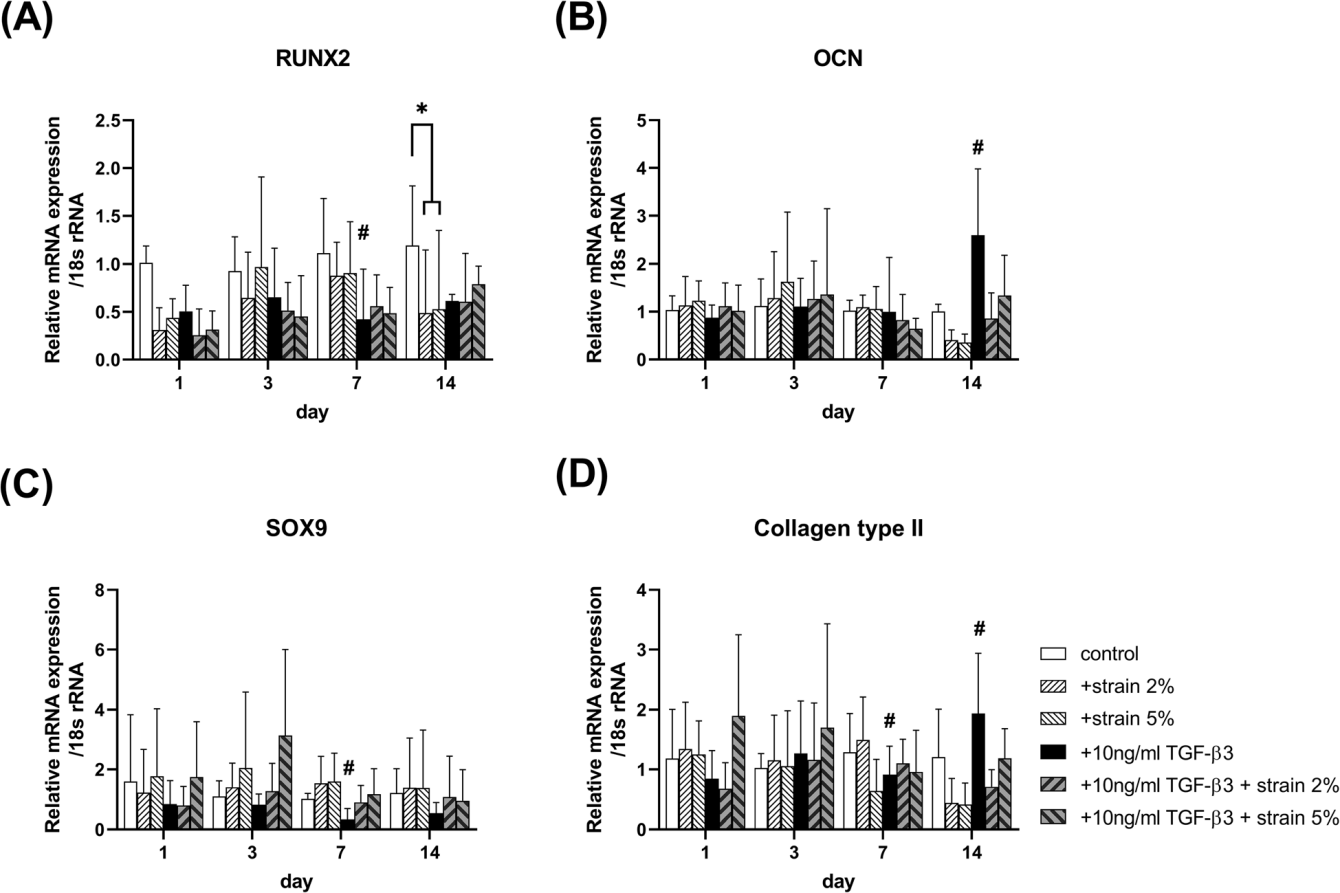
mRNA expression of (**A**) osteogenic genes *RUNX2*, and *OCN*, as well as (**B**) chondrogenic genes *SOX9* and *COL2* measured using qRT-PCR after 1, 3, 7, and 14 days under mechanical strain without or with 10 ng/ml TGF-β3. Data shown are mean fold static condition group ± SEM at each time point

Mechanical stimulation had no effect on the mRNA expression of osteogenic and chondrogenic marker genes in TMSCs, except for runt-related transcription factor 2 (*RUNX2*), at day 14, and there was no change in the expression even under co-treatment with TGF-β3 (Fig. 3).

**Fig. 4:**
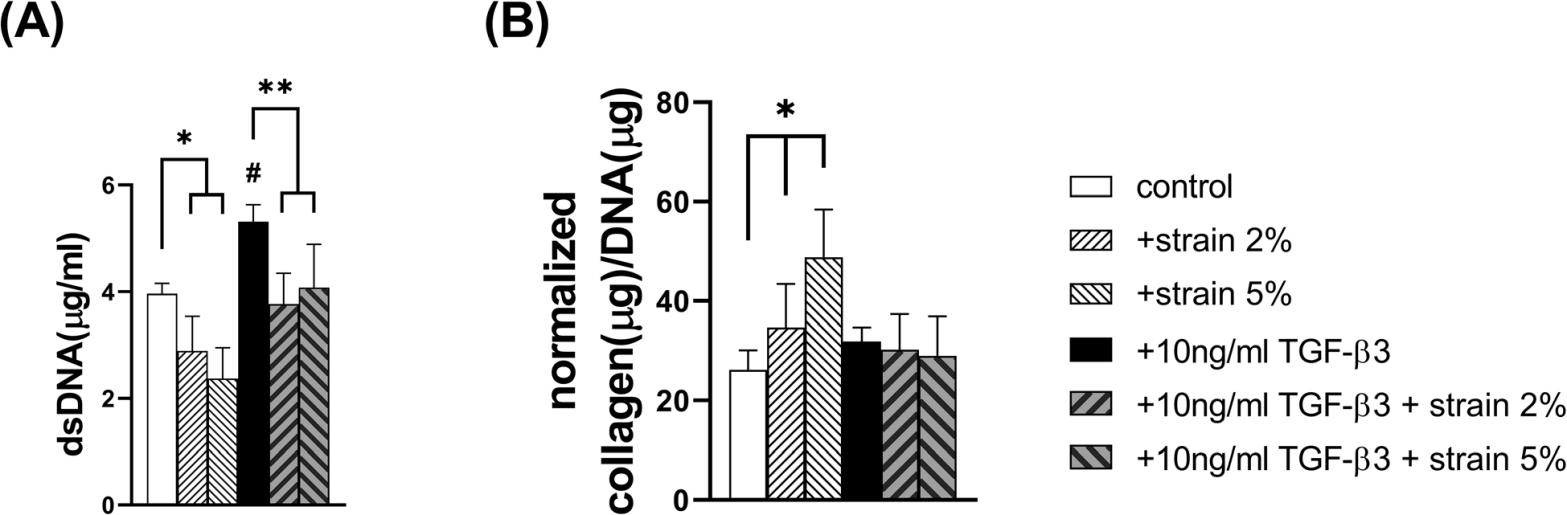
**A** The concentration of dsDNA on day 7 was measured using the PicoGreen® dsDNA Assay Kit upon application of different mechanical strains. **B** The amount of total collagen on day 7 was normalized to dsDNA content upon application of different mechanical strains

To confirm the effect of mechanical stimulation and TGF-β3 on TMSC proliferation, double-stranded DNA (dsDNA) concentration was measured. dsDNA contents were inversely proportional to the intensity of mechanical stimulation in TMSCs. As the intensity of mechanical strain increased, dsDNA concentration decreased, and the total amount of dsDNA increased by TGF-β3 was also decreased by mechanical stimulation (*p < 0.001*; Fig.4A). The total amount of collagen proteins normalized by dsDNA content increased as the intensity of mechanical stimulation without TGF-β3 increased (*p = 0.05* on day 7 and *p < 0.001* on day 14, respectively), but there was no change under simultaneous treatment with TGF-β3 and mechanical stimulation (Fig. 4B).

## Discussion

This study aimed to verify the ability of TGF-β3 and mechanical strain to stimulate the differentiation of TMSCs into tenocytes. We hypothesized that TGF-β3 and mechanical strain induce higher expression of tenocyte-related genes and collagen as end products. TGF-β3 and mechanical strain stimulated TMSC differentiation into tenocytes, although the combination of the two did not induce significantly higher TMSC differentiation.

TMSCs have some advantages over MSCs obtained from other sources. TMSCs are waste tissues from tonsillectomies; thus, they can be obtained via less invasive procedures, as additional surgery is not necessary [15]. Moreover, TMSCs can differentiate more stably than other MSCs, making them suitable for cell banking [17]. Therefore, TMSCs can be easily applied to autografts or allografts [18]. TMSCs have the potential to differentiate into a wide range of tissues. According to previous studies, TMSCs can be osteogenic, adipogenic, chondrogenic, myogenic, and tenogenic [15]. Additionally, TMSCs can be applied in the field of tissue engineering, as the control of the inter- and extracellular environments is possible.

We previously reported that a low concentration of TGF-β3 can induce tenogenic differentiation of TMSCs and increase the expression of *SCX, TNMD*, and *TNC* [16]. Similar results were obtained in this study when comparing the group treated with and without TGF-β3 in the static condition. However, in this study, an increase in the expression of SCX mRNA could not be observed when only TGF-β3 was used to treat TMSCs. Given that the protocol for TMSC stimulation using TGF-β3 was the same for this study and the previous one [16], we thought that the variations might have resulted from the differences in MSC sources. Regarding the expression of SCX, we believe that additional experiments will be required.

We measured the simultaneous effect of TGF-β3 and mechanical strain on TMSCs differentiation into tenocytes. In previous studies, TMSCs were used for chondrogenesis and adipogenesis using a modified 3D scaffold [19–21]. Park et al. reported that a 2D/3D hybrid cell culture system with TGF-β3 increases the expression of chondrogenic genes, such as SRY-Box transcription factor 9 (*SOX9*)*, COL II, COL II A1,* and *COL VII*. Additionally, Patel et al. reported that a composite system of graphene oxide/polypeptide thermogel stimulates the expression of adipogenic genes, such as *PPAR-γ, CEBP-α, LPL, AP2, ELOVL3,* and *HSL*. However, there are insufficient studies regarding the tenogenic expression of TMSCs upon mechanical strain and TGF-β3 treatment, although Yu et al. reported that TGF-β3 induces tenogenesis of TMSCs.

As shown in Fig. 1, the mRNA expression of tenogenic genes, such as *SCX*, was significantly higher when the mechanical strain was applied than under static conditions. SCX is a transcription factor that leads to tenocyte differentiation and suppresses non-tenogenic capacity [22]. These results are similar to those of previous studies in that mechanical strain stimulates the differentiation of MSCs into tenocytes. As shown in Fig. 3, the mRNA expression levels of osteogenic and chondrogenic genes were similar among the static control, 2, and 5% groups. Similarly, Zhang et al. reported that the expression of non-tenocyte-related genes was not significantly altered when mechanical loading was applied to tenocytes [23]. Thus, we believe that mechanical strain can stimulate the differentiation of TMSCs, particularly to tenocytes.

In cells without TGF-β3, dsDNA concentration decreased, while the amount of normalized collagen increased as the intensity of mechanical strain increased. The concentration of dsDNA indicates the quantity of cell proliferation, whereas the amount of collagen as an end product indicates the extent of cell differentiation [24, 25]. The extent of cell proliferation and differentiation is inversely proportional [26]. Therefore, mechanical strain without TGF-β3 stimulates cell differentiation rather than proliferation.

Even though *SCX* was increased with loading and TGF-β3 combinations at days 7 and 14, the two treatments did not have a significant synergistic effect on the expression of other genes. For example, in cells treated with TGF-β3, the mRNA expression level of *COL1, COL3,* and *COL1/3* among mechanical strain groups except *COL3* at day1 had no significant difference. Moreover, under TGF-β3 treatment, mRNA expression was lesser with mechanical strain than without mechanical strain for *TNMD* at day 14, *TNC* at day 14, and *COL3* at day 1. The mRNA expression of *TNC* at day 14 and *COL3* at day 1 decreased when mechanical strain was applied with TGF-β3 treatment, and the mRNA expression of *TNMD* at day 1 and *DCN* without TGF-β3 treatment was higher than that obtained under the combination of TGF-β3 with mechanical strain. Furthermore, in the combination case, the concentration of collagen as an end-product did not significantly change. Although TGF-β3 stimulates TMSC differentiation into tenocytes, the combination of TGF-β3 and mechanical strain does not appear to significantly increase the mRNA expression of tendon-related genes. This result concurs with those of previous studies, which showed that mechanical loading inhibits the differentiation of MSCs with TGF-β3 supplementation [27]. Thorpe et al. reported that continuous dynamic compression from 0 to 42 days in the presence of TGF-β3 inhibits chondrogenesis, whereas delayed dynamic compression after TGF-β3 treatment from 0 to 21 days stimulates chondrogenesis [28]. Therefore, the effect of mechanical loading on MSC differentiation can be manipulated according to the MSC stage.

Furthermore, previous studies have shown that mechanical strain activates the TGF-β3 pathway, which stimulates TMSC differentiation into tenocytes [29]. In this study, TGF-β3 and mechanical strain were treated from the outside of the cell rather than measuring the amount of TGF-β3 expression inside the cell. Hence, this study offers a unique perspective in that the combination of TGF-β3 and mechanical strain did not significantly affect TMSC differentiation. However, the mRNA expression of tenocyte-related genes increased in cells treated with TGF-β3 compared to that in those lacking TGF-β3 treatment. Hence, this study had similar results to previous studies in that TGF-β3 stimulated the differentiation of TMSCs into tenocytes.

This study had some limitations. First, only TGF-β3 was used as a chemical stimulant for differentiation. Other chemicals, such as TGF-β1 or vascular endothelial growth factor, induce TMSC differentiation as well. A combination of TGF-β1 and TGF-β3 treatment can stimulate TMSC differentiation into tenocytes [30]. Second, the effect of TGF-β3 and mechanical strain was only measured for 7 days. Long-term measurements are required to investigate the lasting effect of TGF-β3 and mechanical strain and any possible changes that could occur after a longer duration. Lastly, an immunocytochemistry assay was not conducted. Through immunocytochemistry, the presence of tenogenic proteins as a result of TMSC differentiation was verified [31]. However, in this study, only collagen was measured as the end-product.

## Conclusions

TGF-β3 and mechanical strain stimulate the differentiation of TMSCs, although a combination of the two does not have a significant synergistic effect. Overall, TMSCs have the potential to be used for cell banking, and compared to other MSCs, they can be procured from patients via less invasive procedures. Moreover, TMSCs can differentiate into many types of tissues; thus, they can be applied in the field of tissue engineering to remedy tendon-related diseases without surgery.

## Methods

### Isolation and culture of TMSCs

This study was approved by the Institutional Review Board, and informed consent was obtained from all patients. After tonsillectomy, discarded tonsils were obtained from six patients aged 6–8 years and sectioned into two samples per patient. The tonsil tissues were minced and digested in Dulbecco’s phosphate-buffered saline (Welgene, Daegu, Korea) with 0.075% collagenase type I at 37 °C for 30 min. After filtration through a 100 μm cell strainer, mononuclear cells were obtained from the digested tonsil tissues. The isolated cells were seeded and incubated at a density of 1 × 10^4^ cells/cm^2^ in low-glucose Dulbecco’s modified Eagle’s medium (DMEM; Hyclone, UT, USA), 10% fetal bovine serum (FBS) (Corning, VA, USA), 100 U/mL penicillin, and 100 μg/mL streptomycin (Welgene, Daegu, Korea) in a 5% CO_2_ incubator with humidified air at 37 °C. After 48 h, non-adherent cells were removed, and adherent TMSCs were cultured. We verified the surface markers of MSCs through fluorescence-activated cell sorting using the BD Stemflow™ Human MSC Analysis Kit (BD Biosciences) to confirm the MSC lineage. All TMSCs showed high expression of CD73 (99.93% ± 0.025), CD90 (98.23 ± 1.215), and CD105 (99.08 ± 0.278) and low expression of CD11b, CD19, CD34, CD45, and HLA-DR (0.20 ± 0.041). In addition, the pluripotency of TMSCs was verified using semi-quantitative real-time polymerase chain reaction (PCR) of differentiation marker genes and histological staining for terminal differentiation. TMSCs were harvested for further use between passages 3 and 8.

### TGF-β3 treatment and mechanical strain

TMSCs were subjected to two types of treatment: TGF-β3 and mechanical strain. TMSCs were cultured at 1 × 10^4^ cells/cm^2^ in six-well UniFlex plates coated with COL1. TMSCs were incubated for 1, 3, 7, and 14 days in DMEM/low-glucose containing 10% FBS and 50 μg/mL L-ascorbic acid 2-phosphate (Sigma-Aldrich, MO, USA), with 10 ng/mL TGF-β3 or mechanical strain (R&D Systems, Minneapolis, MN, USA). During harvesting, the medium was changed, and TGF-β3 was supplemented every 2–3 days. The mechanical strain was applied to TMSCs once for 1 h per day using a Flexcell FX-5000 Tension system (Flexcell®, Burlington, NC, USA). The cells were subdivided into three groups: Static, 2, and 5%. The mechanical strain was expressed as a sine wave with a frequency of 0.5. Each experiment was repeated twice per patient.

### Quantitative real-time PCR (qRT-PCR)

Total RNA was extracted and reverse-transcribed using a first-strand cDNA synthesis kit (Invitrogen, Waltham, MA, USA). qRT-PCR was performed using the SensiFAST™ SYBR® Hi-ROX kit (Bioline, London, UK) according to the manufacturer’s instructions. mRNA expression levels were estimated using the Green I dye. Each specific primer was designed using Nucleotide BLAST, and 18S ribosomal RNA was used as an internal control (Table 1).

**Table 1:** Primer sequences used in this study to measure specific gene expression

Primer	Forward 5′-3′ Sequence	Reverse 5′-3′ Sequence
*18 s rRNA*	GTAACCCGTTGAACCCCATT	CCATCCAATCGGTAGTAGCG
*SCX*	ACAGATCTGCACCTTCTGCC	GCCACCTCCTAACTGCGAAT
*TNMD*	TCCCTCAGGCTCTGGTATGA	AGGACTGAGAGACCACTGCT
*DCN*	TGCCAAAGGATCTTCCCCCT	AGGTGTAAATGCTCCAGGACT
*COL1*	AGTGGTTTGGATGGTGCCAA	GCACCATCATTTCCACGAGC
*COL3*	TGGAGGATGGTTGCACGAAA	ACAGCCTTGCGTGTTCGATA
*TNC*	ATGGGCAGACGCACCATTAG	TGTGCATCGACCTTCACAAGA
*OCN*	TCCTTTGGGGTTTGGCCTAC	CCAGCCTCCAGCACTGTTTA
*RUNX2*	CCTACCTGAGCCAGATGACG	ATGCTGGGTGGCCTGAAAT
*COL2*	GCTCCTGCCGTTTCGCTG	ATTATACCTCTGCCCATCCTGC
*SOX9*	AGGAAGTCGGTGAAGAACGG	AAGTCGATAGGGGGCTGTCT

### DNA content assay

dsDNA concentration was measured on day 7 to assess cell proliferation under each condition and normalize collagen content as an end product using the Quant-iT™ PicoGreen®dsDNA Assay Kit (Invitrogen, Waltham, MA, USA) according to the manufacturer’s instructions. The fluorescence intensity of the samples was measured to assess dsDNA concentration, whereby cells were digested in 1.5 mL of 0.15 mg/mL papain extraction reagent (Sigma-Aldrich, MO, USA) for 3 h at 65 °C and excited at 485 nm, and the emittance was measured at 538 nm.

### Collagen assay

Collagen concentration was assessed on day 7 using the Sircol™ Collagen Assay Kit (Biocolor, Antrim, UK). For collagen isolation, 2 mL of 0.1 mg/mL pepsin in 0.5 M acetic acid was added to the cultivated media. Pepsin digestion was performed overnight at 4 °C with constant agitation of the medium. To concentrate the collagen sample, 100 μL of acid-neutralizing reagent was added to 1 mL of the samples and cell debris was removed by centrifugation. As a blank control, 100 μL of cold isolation and concentration reagent was added to all samples, incubated overnight at 4 °C, and centrifuged at 13,000×g for 10 min, after which the supernatant was discarded. One milliliter of Sircol dye reagent was added to the samples and incubated at room temperature for 30 min with constant agitation. After centrifugation, the supernatant was discarded, the pellet of each sample was dissolved in 1 mL of alkali reagent, and the absorbance was measured at 550 nm.

### Statistical analysis

mRNA expression data were normalized to 0% strain value without TGF-β3 for each time. To compare the tenogenic effect of TGF-β3 in TMSCs, the non-TGF-treated group and the treated group without mechanical stimulation were statistically analyzed with an independent sample t-test. Statistical analysis among each group on days 1, 3, 7, and 14 was performed using one-way analysis of variance (ANOVA) and Tukey’s post-hoc test using SPSS Statistics (SPSS, Inc., Chicago, IL, USA). A representative graph was constructed using GraphPad Prism 8 software (GraphPad Software Inc., La Jolla, CA, USA). Significant differences are represented as follows (*p < 0.05)*: static control vs strain groups without TGF-β3(*), static control with TGF-β3 vs other groups (**), and without vs with 10 ng/ml TGF-β3 on static control (#).

## Data Availability

Not applicable.

## Abbreviations

COL1: Collagen type 1
COL2: Collagen type 2
COL3: Collagen type 3
DCN: Decorin
dsDNA: Double-stranded DNA
DMEM-LG: Low-glucose Dulbecco’s modified Eagle’s medium
ECM: Extracellular matrix
FBS: Fetal bovine serum
MSCs: Mesenchymal stem cells
qRT-PCR: Quantitative real-time polymerase chain reaction
RUNX2: Runt-related transcription factor 2
SCX: Scleraxis
SOX9: SRY-Box transcription factor 9
TGF-β3: Transforming growth factor-beta 3
TMSCs: Tonsil-derived mesenchymal stem cells
TNC: Tenascin-C
TNMD: Tenomodulin
