# Correction to: Influence of mechanical and TGF-β3 stimulation on the tenogenic differentiation of tonsil-derived mesenchymal stem cells

**DOI:** 10.1186/s12860-022-00406-9

**Published:** 2022-01-28

**Authors:** Jaeyeon Wee, Hyang Kim, Sang-Jin Shin, Taeyong Lee, Seung Yeol Lee

**Affiliations:** 1 School of Medicine, CHA University, Pocheon, South Korea; 2 New Horizon Biomedical Engineering Institute, Myongji Hospital, Goyang, South Korea; 3 Department of Orthopaedic Surgery, Ewha Womans University Seoul Hospital, Seoul, South Korea; 4 Division of Mechanical and Biomedical Engineering, Ewha Womans University, Seoul, South Korea; 5 Department of Orthopaedic Surgery, Myongji Hospital, Hanyang University College of Medicine, 55, Hwasu-ro 14beon-gil, Deogyang-gu, Goyang, Gyeonggi 10475 South Korea

**Correction to: BMC Mol Cell Biol 23, 3 (2022)**

**https://doi.org/10.1186/s12860-021-00400-7**

Following publication of the original article [1], a typesetting error was noticed in the reference list. References 17 to 31 were published in the incorrect order. The correct order of references 17 to 31 are given below and the original article [1] has been corrected. The publishers apologize for the error.

17. Ryu KH, Cho KA, Park HS, Kim JY, Woo SY, Jo I, et al. Tonsil-derived mesenchymal stromal cells: evaluation of biologic, immunologic and genetic factors for successful banking. Cytotherapy. 2012;14(10):1193 –202. 10.3109/14653249.2012.706708 .

18. Choi JS, Lee BJ, Park HY, Song JS, Shin SC, Lee JC, et al. Effects of donor age, long-term passage culture, and cryopreservation on tonsil-derived mesenchymal stem cells. Cell Physiol Biochem. 2015;36(1):85 –99. 10.1159/000374055.

19. Koh RH, Jin Y, Kang BJ, Hwang NS. Chondrogenically primed tonsil-derived mesenchymal stem cells encapsulated in riboflavin-induced photocrosslinking collagen-hyaluronic acid hydrogel for meniscus tissue repairs. Acta Biomater. 2017;53:318–28. 10.1016/j.actbio.2017.01.081.

20. Park J, Kim IY, Patel M, Moon HJ, Hwang SJ, Jeong B. 2D and 3D hybrid Systems for Enhancement of chondrogenic differentiation of tonsil-derived mesenchymal stem cells. Adv Funct Mater. 2015;25(17):2573–82. 10.1002/adfm.201500299.

21. Patel M, Moon HJ, Ko DY, Jeong B. Composite system of graphene oxide and polypeptide thermogel as an injectable 3D scaffold for adipogenic differentiation of tonsil-derived mesenchymal stem cells. ACS Appl Mater Interfaces. 2016;8(8):5160–9. 10.1021/acsami.5b12324.

22. Li YH, Ramcharan M, Zhou ZP, Leong DJ, Akinbiyi T, Majeska RJ, et al. The role of Scleraxis in fate determination of mesenchymal stem cells for tenocyte differentiation. Sci Rep-Uk. 2015;5(1). 10.1038/srep13149.

23. Zhang JY, Wang JHC. The effects of mechanical loading on tendons - an in vivo and in vitro model study. Plos One. 2013;8(8).

24. Jager M, Feser T, Denck H, Krauspe R. Proliferation and osteogenic differentiation of mesenchymal stem cells cultured onto three different polymers in vitro. Ann Biomed Eng. 2005;33(10):1319–32. 10.1007/s10439-005-5889-2.

25. Fan HB, Liu HF, Toh SL, Goh JCH. Enhanced differentiation of mesenchymal stem cells co-cultured with ligament fibroblasts on gelatin/silk fibroin hybrid scaffold. Biomaterials. 2008;29(8):1017–27. 10.1016/j.biomaterials.2007.10.048.

26. Agathocleous M, Harris WA. Metabolism in physiological cell proliferation and differentiation. Trends Cell Biol. 2013;23(10):484–92. 10.1016/j.tcb.2013.05.004.

27. Lima EG, Bian L, Ng KW, Mauck RL, Byers BA, Tuan RS, et al. The beneficial effect of delayed compressive loading on tissue-engineered cartilage constructs cultured with TGF-beta 3. Osteoarthr Cartil. 2007;15(9):1025–33. 10.1016/j.joca.2007.03.008.

28. Thorpe SD, Buckley CT, Vinardell T, O'Brien FJ, Campbell VA, Kelly DJ. The response of bone marrow-derived mesenchymal stem cells to dynamic compression following TGF-beta 3 induced Chondrogenic differentiation. Ann Biomed Eng. 2010;38(9):2896–909. 10.1007/s10439-010-0059-6

29. Nam HY, Pingguan-Murphy B, Abbas AA, Merican AM, Kamarul T. Uniaxial cyclic tensile stretching at 8% strain exclusively promotes Tenogenic differentiation of human bone marrow-derived mesenchymal stromal cells. Stem Cells Int. 2019;2019:1 –16. 10.1155/2019/9723025 .

30. Bottagisio M, Lopa S, Granata V, Talo G, Bazzocchi C, Moretti M, et al. Different combinations of growth factors for the tenogenic differentiation of bone marrow mesenchymal stem cells in monolayer culture and in fibrin-based three-dimensional constructs. Differentiation. 2017;95:44–53. 10.1016/j.diff.2017.03.001.

31. Kraus A, Woon C, Raghavan S, Megerle K, Pham H, Chang J. Co-culture of human adipose-derived stem cells with tenocytes increases proliferation and induces differentiation into a tenogenic lineage. Plast Reconstr Surg. 2013;132(5):754e –66e
